# A Nomogram for Predicting the Risk of Bone Metastasis in Newly Diagnosed Head and Neck Cancer Patients: A Real-World Data Retrospective Cohort Study From SEER Database

**DOI:** 10.3389/fgene.2022.865418

**Published:** 2022-05-30

**Authors:** Chao Huang, Jialin He, Zichuan Ding, Hao Li, Zongke Zhou, Xiaojun Shi

**Affiliations:** ^1^ Department of Orthopedics, West China Hospital of Sichuan University, Chengdu, China; ^2^ Department of Orthopedics, The Second Affiliated Hospital of Zunyi Medical University, Zunyi, China

**Keywords:** head and neck cancer, nomogram, risk, bone metastasis, SEER

## Abstract

**Background:** Bone metastasis (BM) is one of the typical metastatic types of head and neck cancer (HNC). The occurrence of BM prevents the HNC patients from obtaining a long survival period. Early assessment of the possibility of BM could bring more therapy options for HNC patients, as well as a longer overall survival time. This study aims to identify independent BM risk factors and develop a diagnostic nomogram to predict BM risk in HNC patients.

**Methods:** Patients diagnosed with HNC between 2010 and 2015 were retrospectively evaluated in the Surveillance, Epidemiology, and End Results (SEER) database, and then eligible patients were enrolled in our study. First, those patients were randomly assigned to training and validation sets in a 7:3 ratio. Second, univariate and multivariate logistic regression analyses were used to determine the HNC patients’ independent BM risk factors. Finally, the diagnostic nomogram’s risk prediction capacity and clinical application value were assessed using calibration curves, receiver operating characteristic (ROC), and decision curve analysis (DCA) curves.

**Results:** 39,561 HNC patients were enrolled in the study, and they were randomly divided into two sets: training (*n* = 27,693) and validation (*n* = 11,868). According to multivariate logistic regression analysis, race, primary site, tumor grade, T stage, N stage, and distant metastases (brain, liver, and lung) were all independent risk predictors of BM in HNC patients. The diagnostic nomogram was created using the above independent risk factors and had a high predictive capacity. The training and validation sets’ area under the curves (AUC) were 0.893 and 0.850, respectively. The AUC values of independent risk predictors were all smaller than that of the constructed diagnostic nomogram. Meanwhile, the calibration curve and DCA also proved the reliability and accuracy of the diagnostic nomogram.

**Conclusion:** The diagnostic nomogram can quickly assess the probability of BM in HNC patients, help doctors allocate medical resources more reasonably, and achieve personalized management, especially for HNC patients with a potentially high BM risk, thus acquiring better early education, early detection, and early diagnosis and treatment to maximize the benefits of patients.

## Introduction

According to the latest GLOBOCAN 2020 compiled by the International Agency for Research on Cancer, head and neck cancer (HNC) is the seventh most prevalent malignant tumor globally. There were approximately 932,000 new HNC patients worldwide in 2020, of which 467,000 patients had died (Sung et al., 2021). Although in the past 40 years, with the continuous developments in detection and treatment technologies, the 5-years survival rate of HNC has grown from 54.1% to 66.8%, but it still cannot meet the needs of patients for a more extended survival period (Guo et al., 2021). The incidence of distant metastases from HNC ranges from 8.9–13.8%, and distant metastases is one of the main challenges that restrict the successful treatment of HNC patients (Lee et al., 2012; Duprez et al., 2017). Among them, bone metastasis (BM) is the second most prevalent type of distant metastases after lung metastasis, accounting for about 15–39% of all distant metastases’ patients, which leads to a poor prognosis and seriously affects life quality of those patients (Suzuki et al., 2020). Since the previous studies have shown that more than 90% of the histological types of HNC were related to squamous cell carcinomas (SCC), even under the best systemic treatment, the median overall survival (OS) of patients with metastatic SCC was only 10.1 months (Mourad et al., 2017), while patients with multi-organ or polyostotic metastases had a median survival time of fewer than 5.7 months (Suzuki et al., 2020). According to reports, the proportion of HNC patients who died of distant metastases was about 15–20%, but the autopsy series of observations revealed distant metastases was 3–4 times greater than those described in the clinical series (Duprez et al., 2017). Early diagnosis of BM is essential to avoid skeletal-related events with altered performance status and reduce the chance of receiving adequate systemic treatment. In the actual treatment process, due to the insidious nature of distant metastases, most of them were easily overlooked or missed, making many patients already have advanced metastasis with skeletal-related events by the time they arrive at the hospital and need to take adequate systemic treatment. Therefore, effective prediction of the risk of distant metastases in HNC patients is essential to ensure the best benefit for patients.

Currently, the prognosis of HNC patients with BM and the OS prediction of HNC patients have been described (Carvalho et al., 2005; Mourad et al., 2017; Chi et al., 2021). However, to our knowledge, there are few studies based on big data to explore which factors cause BM in HNC patients and to establish effective risk prediction models to predict the risk of BM in newly diagnosed HNC patients, which is crucial in the intervention and treatment of early BM. Therefore, we developed a diagnostic nomogram model based on the SEER database for predicting the risk of BM in HNC patients, aiming to assist clinicians to manage HNC patients better, detect and intervene in BM early, and effectively prolong the survival period of HNC patients, especially those who are at a greater risk of developing BM.

## Methods

### Database

The SEER database is a cancer database based on nearly 30% of the population of the United States of America. It includes demographic and clinical pathology information on cancer incidence and survival rates from 18 cancer registries (Lin et al., 2018). After obtaining permission to access the research data with reference number 16336-Nov 2020, we gained the SEER database. The included patients were those diagnosed as HNC with or without BM from 2010 to 2016 in the SEER database obtained through SEER Stat 8.3.9.2 [Incidence-SEER 18 Regs Custom Data (with additional treatment fields), November 2018 Sub (1975–2016 varying)]. The SEER database is an open-access database, and the data information obtained is anonymous and de-identified; therefore, the ethics committee’s approval and the informed consent of the patients do not apply to this study. Moreover, this study was conducted and reported following the STROCSS 2019 criteria (Riaz et al., 2019).

### Patient Selection

The following were the criteria for inclusion: (1): HNC was the first or primary tumor; (2); the patient’s ICD-O-3 histological type was clear; (3); distant metastases (bone, liver, brain, and lung) was evident, especially bone; (4); complete follow-up data. Meanwhile, the following were the exclusion criteria: (1): HNC was not the first or primary tumor; (2); unclear histological classification; (3); information about distant metastases (bone, liver, brain, and lung), race, tumor grade, T stage, N stage, insurance status, and marital status was unknown; (4); the survival time was less than 1 month. Finally, a total of 39,561 HNC patients were enrolled in this study.

### Variable Definitions

From the SEER database, we retrieved 13 characteristics that may be related to the development of BM in HNC patients. Age, sex, race, insurance status, and marital status were among the demographic characteristics studied. Age was divided into ≥60 and <60 (Han et al., 2021; Sarfraz et al., 2021; Tadros et al., 2021; Zhou et al., 2021), sex was divided into male and female; race was divided into black, white, and other; single, unmarried or domestic partner, widowed, separated, and divorced were classified as the unmarried group, while the insured/no specifics, insured, and any Medicaid were classified as the insured group. Tumor features included tumor grade (I, II, III, and IV), T stage (T1, T2, T3, and T4), N stage (N0, N1, N2, and N3); the histological type was divided into SCC and other. Distant metastases (bone, liver, brain, and lung) were divided into present and absent.

### Statistical Analysis

SPSS (version 22.0) and R software (version 4.0.3) were used to conduct all statistical analyses in this study. Furthermore, a *p*-value <0.05 was considered to be statistically different. These included 39561 patients were randomly separated into a training set (n = 27693, 70%) and a validation set (*n* = 11868, 30%) in a 7:3 ratio using R software. We used the training set to evaluated the independent risk predictors of BM in HNC patients, constructed a diagnostic nomogram model, and verified the constructed nomogram using the validation set. Specifically, we used the training set to perform univariate logistic analysis in SPSS to determine the risk factors related to BM. The variables with a *p*-value <0.05 in the univariate logistic regression analysis were further incorporated into the multivariate logistic regression analysis to determine the independent risk predictors of BM in HNC patients. Then, those independent risk predictors were used to construct a diagnostic nomogram model using R software, and the corresponding score assignment of independent risk predictors was obtained (Supplementary Table S1). After that, a calibration curve was constructed to show the diagnostic nomogram’s correction ability. Then, a receiver operating characteristic (ROC) curve was performed, and the area under the curve (AUC) was used to indicate the diagnostic nomogram’s discrimination. Furthermore, we used the previously obtained scores assigned to independent risk predictors to calculate the patient’s total score and draw ROC curves by SPSS and R software to gain and compare the AUC of each independent risk factor and the diagnostic nomogram. Finally, a decision curve analysis (DCA) was constructed to assess the diagnostic nomogram’s clinical applicability utility.

## Results

### Baseline Characteristics

According to our inclusion and exclusion criteria, 39,561 HNC patients from 2010 to 2015 were finally included in the study, with 27,693 patients in the training set and the remaining 11,868 patients in the validation set. The goal of the study was to explore and verify independent risk predictors of BM in HNC patients. Patients in the training set showed male and white bias characteristics, accounting for 73.3% and 82.9% of the total patients, respectively. The majority of patients were insured (95.5%), while age and marital status had no significant difference. The primary common histological type of HNC patients was SCC (90.1%), and the top three primary sites were oral cavity (38.0%), larynx (24.0%), and oropharynx (18.1%). The most prevalent tumor grade, T stage, and N stage were grade II (48.8%), T2 (29.2%), and N0 (50.7%), respectively. At the same time, patients with distant metastases accounted for a small number of patients. The baseline information for all patients is shown in Table 1.

**TABLE 1:** The clinical and pathological characteristics of the 39,561 individuals with head and neck cancer who were enrolled in our retrospective cohort analysis.

Variables	Training cohort (27,693)				Validation cohort (11,868)			
	Without BM		With BM		Without BM		With BM	
	27,445	99.10 (%)	248	0.90 (%)	11,784	99.29 (%)	84	0.71 (%)
Age (years)								
<60	11,906	43.38	114	45.97	5,142	43.64	36	42.86
≥60	15,539	56.62	134	54.03	6,642	56.36	48	57.14
Sex								
Male	20,103	73.25	189	76.21	8,664	73.52	63	75.00
Female	7,342	26.75	59	23.79	3,120	26.48	21	25.00
Race								
Black	2,836	10.33	48	19.35	1,254	10.64	21	25.00
White	22,785	83.02	171	68.95	9,789	83.07	56	66.67
Other	1,824	6.65	29	11.69	741	6.29	7	8.33
Marital status								
Unmarried	12,274	44.72	126	50.81	5,278	44.79	46	54.76
Married	15,171	55.28	122	49.19	6,506	55.21	38	45.24
Insurance status								
No	1,233	4.49	25	10.08	537	4.56	10	11.90
Yes	26,212	95.51	223	89.92	11,247	95.44	74	88.10
Histological types								
Squamous cell carcinoma	24,751	90.18	203	81.85	10,630	90.21	71	84.52
Others	2,694	9.82	45	18.15	1,154	9.79	13	15.48
Primary site								
Oral cavity	10,466	38.13	71	28.63	4,463	37.87	31	36.90
Lip	852	3.10	1	0.40	360	3.05	0	0.00
Oropharynx	4,954	18.05	46	18.55	2,177	18.47	8	9.52
Nasopharynx	733	2.67	43	17.34	319	2.71	13	15.48
Hypopharynx	1,004	3.66	15	6.05	425	3.61	4	4.76
Salivary gland	1,962	7.15	30	12.10	855	7.26	7	8.33
Sinonasal	843	3.07	17	6.85	388	3.29	7	8.33
Larynx	6,631	24.16	25	10.08	2,797	23.74	14	16.67
Tumor grade								
Grade Ⅰ	4,341	15.82	7	2.82	1,806	15.33	5	5.95
Grade Ⅱ	13,433	48.95	74	29.84	5,894	50.02	26	30.95
Grade Ⅲ	8,928	32.53	146	58.87	3,771	32.00	42	50.00
Grade Ⅳ	743	2.71	21	8.47	313	2.66	11	13.10
T Stage								
T1	9,275	33.79	31	12.50	3,996	33.91	14	16.67
T2	8,053	29.34	44	17.74	3,454	29.31	16	19.05
T3	4,758	17.34	56	22.58	2,085	17.69	13	15.48
T4	5,359	19.53	117	47.18	2,249	19.09	41	48.81
N Stage								
N0	13,995	50.99	45	18.15	6,013	51.03	15	17.86
N1	3,907	14.24	59	23.79	1,715	14.55	16	19.05
N2	8,819	32.13	121	48.79	3,745	31.78	50	59.52
N3	724	2.64	23	9.27	311	2.64	3	3.57
Lung metastasis								
Absent	27,079	98.67	172	69.35	11,630	98.69	63	75.00
Present	366	1.33	76	30.65	154	1.31	21	25.00
Liver metastasis								
Absent	27,373	99.74	202	81.45	11,754	99.75	73	86.90
Present	72	0.26	46	18.55	30	0.25	11	13.10
Brain metastasis								
Absent	27,424	99.92	235	94.76	11,778	99.95	81	96.43
Present	21	0.08	13	5.24	6	0.05	3	3.57

### Identification of Independent Risk Predictors of Bone Metastasis in HNC Patients

Of the 39,561 HNC patients, 332 (0.8%) patients were diagnosed with BM, while 39,229 (99.2%) HNC patients were diagnosed without BM. Univariate logistic analysis was performed by incorporating the 13 variables. The results showed that race, insurance status, histological type, primary site, tumor grade, T stage, N stage, and distant metastases (brain, liver, and lung) were associated with the development of BM in HNC patients (*p* < 0.05) (Table 2). Then, based on the variables mentioned above, multivariate logistic regression analysis revealed that race, primary site, tumor grade, T stage, N stage, and distant metastases (brain, liver, and lung) were independent risk predictors of BM in HNC patients (Table 2). In other words, black HNC patients with nasopharynx lesion, grade III, T4 stage, and N3 stage, and distant metastases (brain, liver, and lung) had a high risk of BM.

**TABLE 2:** The logistic regression analysis of independent risk factors of bone metastasis in head and neck cancer patients in our retrospective cohort study.

Variables	Univariate analysis		Multivariate analysis	
	OR (95% CI)	*p* value	OR (95% CI)	*p* value
Age (years)				
<60	Reference			
≥60	0.901 (0.701–1.157)	0.413		
Sex				
Male	Reference			
Female	0.855 (0.637–1.146)	0.295		
Race				
Black	Reference		Reference	
White	0.443 (0.321–0.612)	<0.001	0.588 (0.409–0.847)	0.004
Other	0.939 (0.590–1.495)	0.792	0.605 (0.346–1.058)	0.078
Marital status				
Unmarried	Reference			
Married	0.783 (0.610–1.006)	0.056		
Insurance status				
No	Reference			
Yes	0.420 (0.276–0.637)	<0.001		
Histological type				
Squamous cell carcinoma	Reference			
Others	2.037 (1.471–2.820)	<0.001		
Primary site				
Oral cavity	Reference		Reference	
Lip	0.173 (0.024–1.247)	0.082	0.609 (0.081–4.575)	0.630
Oropharynx	1.369 (0.943–1.987)	0.099	0.901 (0.600–1.351)	0.613
Nasopharynx	8.647 (5.878–12.722)	<0.001	4.356 (2.964–7.042)	<0.001
Hypopharynx	2.202 (1.257–3.858)	0.006	0.761 (0.403–1.440)	0.402
Salivary gland	2.254 (1.467–3.463)	<0.001	1.921 (1.173–3.145)	0.009
Sinonasal	2.973 (1.743–5.070)	<0.001	2.596 (1.375–4.902)	0.003
Larynx	0.556 (0.352–0.878)	0.012	0.576 (0.354–0.936)	0.026
Tumor grade				
Grade Ⅰ	Reference		Reference	
Grade Ⅱ	3.416 (1.573–7.421)	0.002	1.952 (0.878–4.340)	0.101
Grade Ⅲ	10.141 (4.746–21.668)	<0.001	3.806 (1.728–8.383)	0.001
Grade Ⅳ	17.528 (7.425–41.376)	<0.001	3.121 (1.226–7.945)	0.017
T Stage				
T1	Reference		Reference	
T2	1.635 (1.031–2.591)	0.036	1.020 (0.621–1.673)	0.939
T3	3.521 (2.268–5.469)	<0.001	1.789 (1.108–2.890)	0.017
T4	6.532 (4.390–9.719)	<0.001	3.071 (1.984–4.753)	<0.001
N Stage				
N0	Reference		Reference	
N1	4.696 (3.181–6.933)	<0.001	2.375 (1.522–3.705)	<0.001
N2	4.267 (3.027–6.015)	<0.001	2.361 (1.581–3.526)	<0.001
N3	9.880 (5.945–16.418)	<0.001	3.273 (1.790–5.985)	<0.001
Lung metastasis				
Absent	Reference		Reference	
Present	32.692 (24.487–43.646)	<0.001	12.710 (8.976–17.997)	<0.001
Liver metastasis				
Absent	Reference		Reference	
Present	86.576 (58.325–128.511)	<0.001	18.885 (11.718–30.436)	<0.001
Brain metastasis				
Absent	Reference		Reference	
Present	72.242 (35.749–145.986)	<0.001	9.278 (3.705–23.235)	<0.001

### Establishment and Verification of the Diagnostic Nomogram for BM in HNC Patients

**FIGURE 1:**
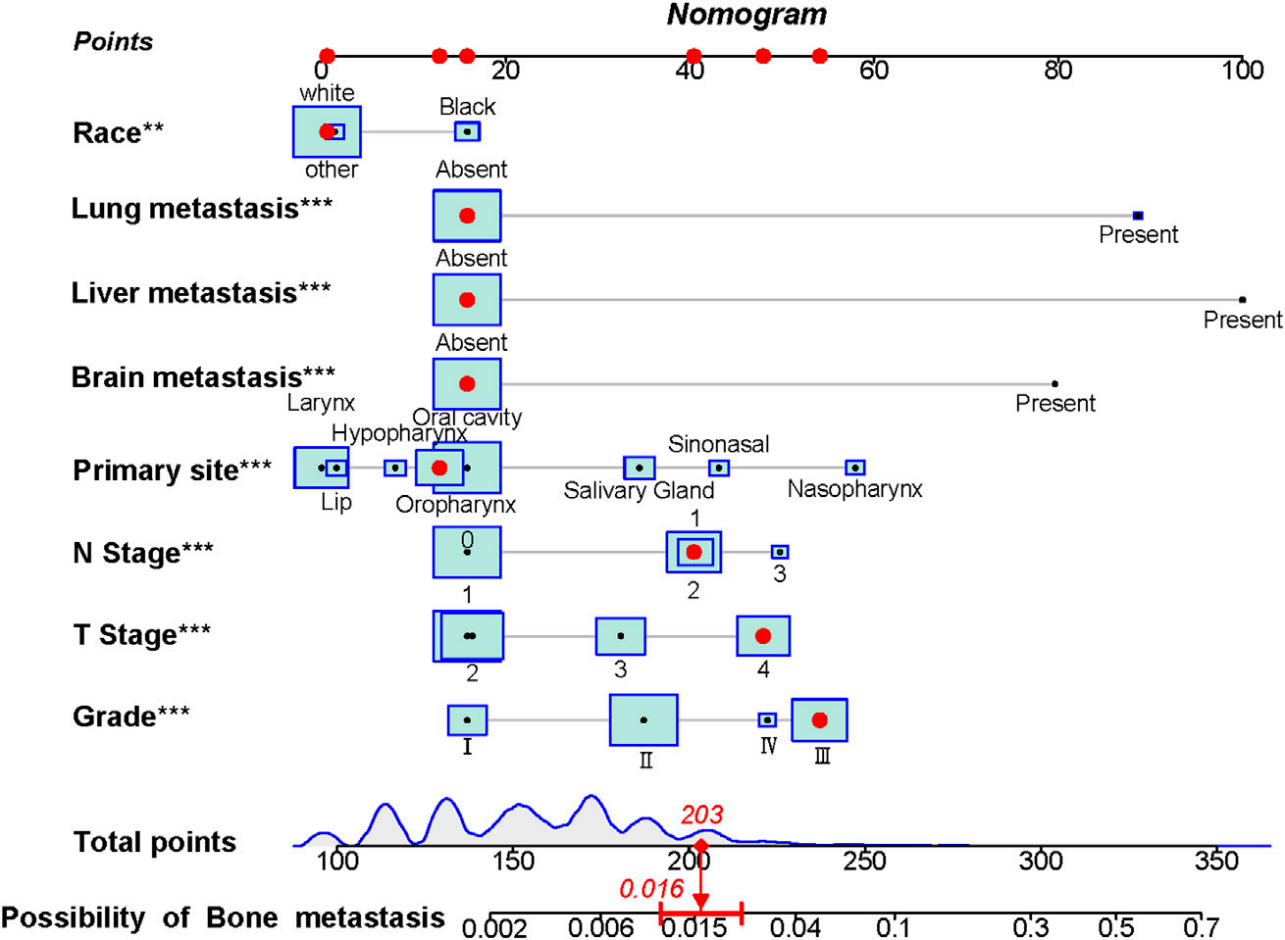
The nomogram was used to predict the risk of bone metastasis in head and neck cancer patients. Each independent risk factor predicting the occurrence of BM in an individual patient is located on the left side of the nomogram, and its corresponding point is located on the variable axis above, with a line drawn upward to the point axis to determine the number of points assigned to each independent risk factor. A total point line is located at the bottom of the nomogram, and the points corresponding to each independent variable are summed to give a total point. Then, a vertical line is drawn from the total point scale to the BM axis to obtain the probability of BM. For example, a patient of white race has no distant metastasis (liver, brain, lung), and the primary site is in the oropharynx with N1 stage, T4 stage, and Grade III. The corresponding total points of this patient’s is 1 (white race) + 16 (no lung metastasis) + 16 (no liver metastasis) + 16 (no brain metastasis) + 13 (oropharynx site) + 40 (N1 stage) + 48 (T4 stage) + 53 (Grade III) = 203, and this patient’ corresponding risk possibility of BM is 0.016.

**FIGURE 2:**
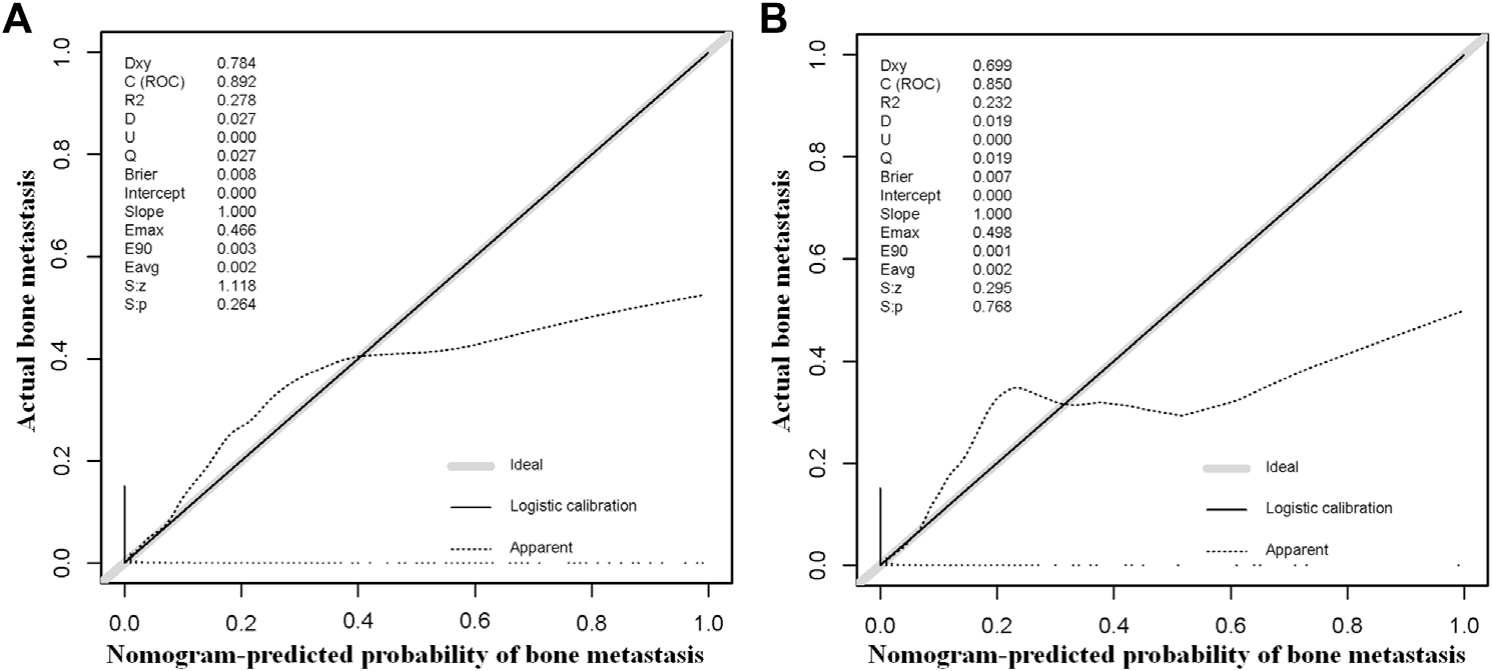
The training and the validation sets’ calibration curves of the constructed nomogram in our study were used to predict the risk of bone metastasis in head and neck cancer patients. Grey line denotes ideal; black line logistic calibration; dotted line apparent.

**FIGURE 3:**
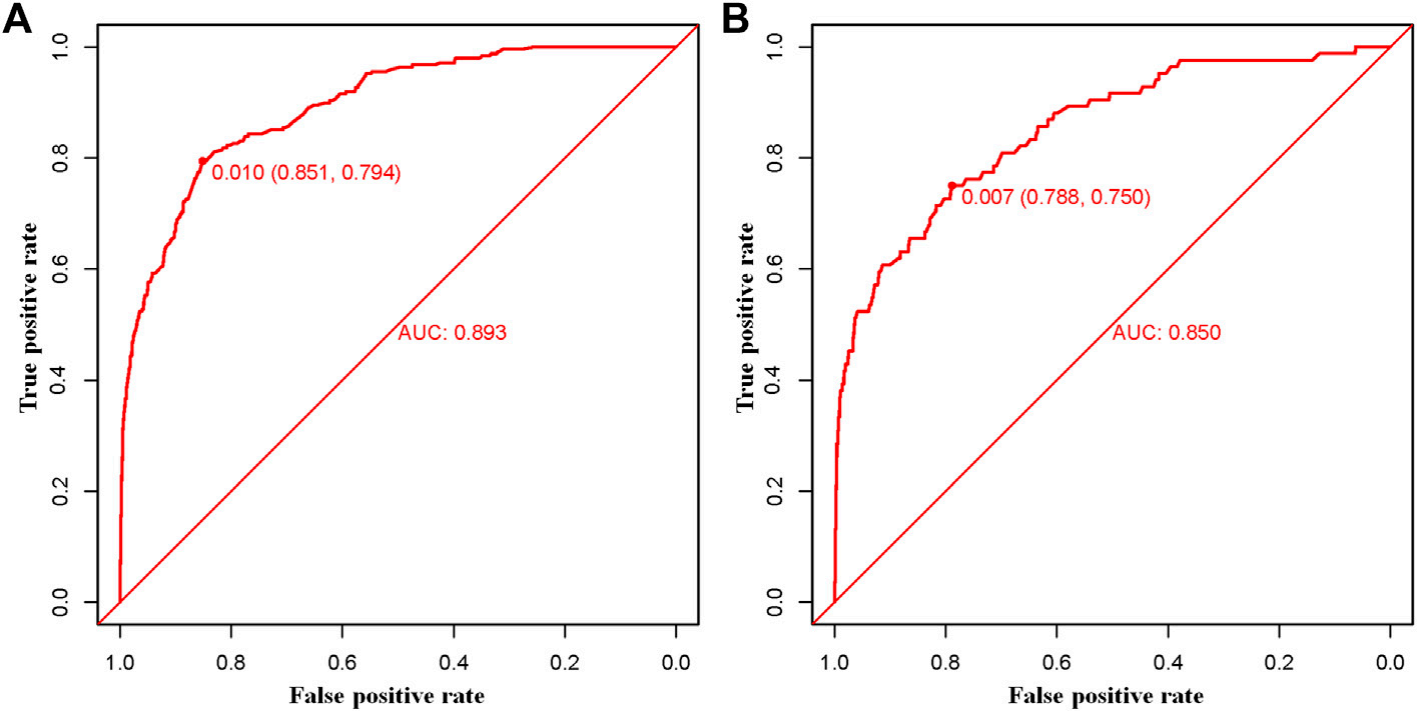
The training **(A)** and the validation **(B)** sets’ receiver operating characteristic (ROC) curve and area under the curve (AUC) of the constructed nomogram were used to predict bone metastasis of head and neck cancer patients.

**FIGURE 4:**
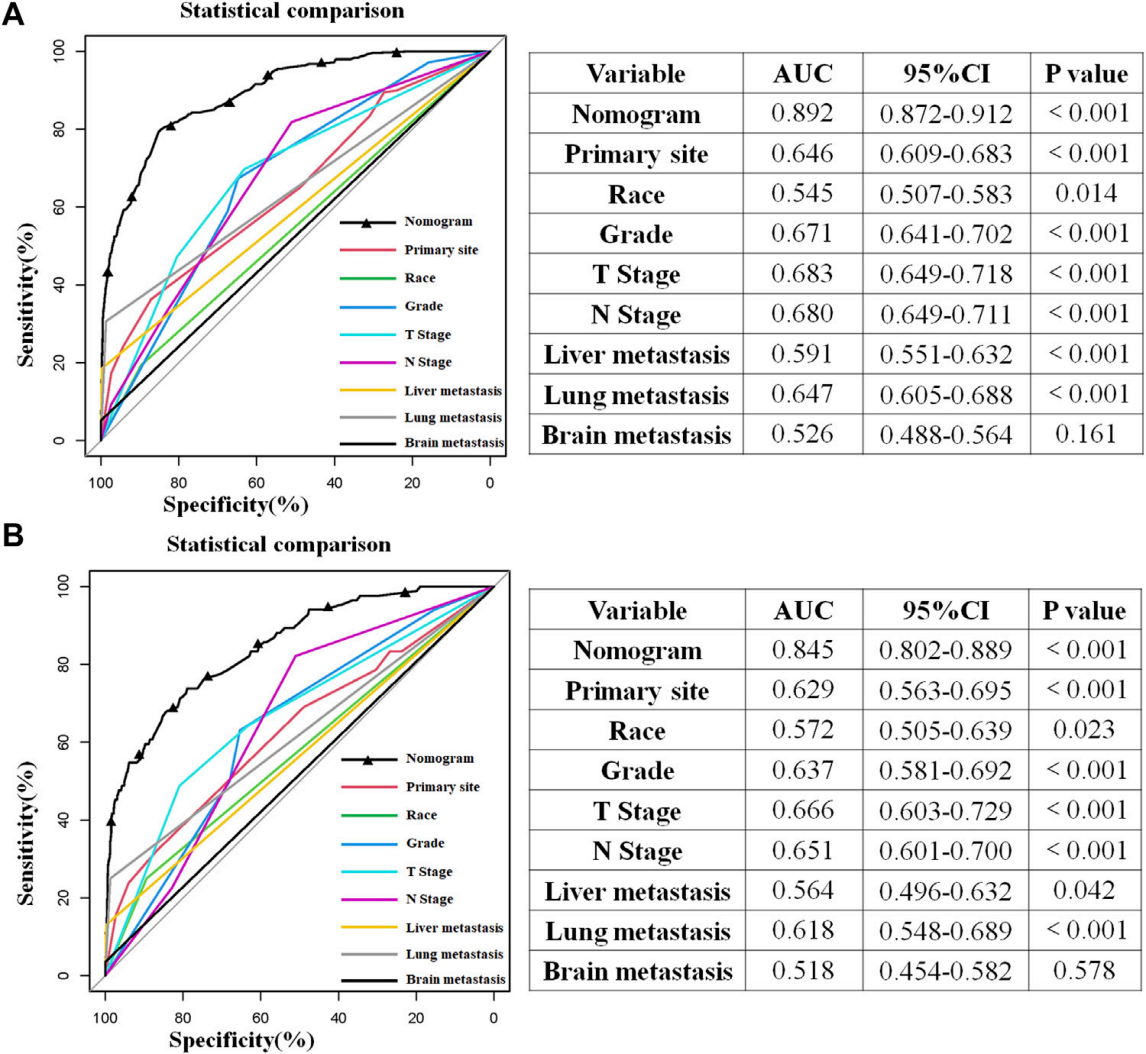
Comparison of the area under the receiver operating characteristic curve between the constructed nomogram and the independent predictors in the training **(A)** and test sets **(B)**. Moreover, the AUC’s results built by SPSS software show that the combined model had the highest AUC in the training **(A)** and test sets **(B)**, which showed that the constructed nomogram had excellent predictive ability in predicting the probability of bone metastasis in patients with head and neck cancer.

**FIGURE 5:**
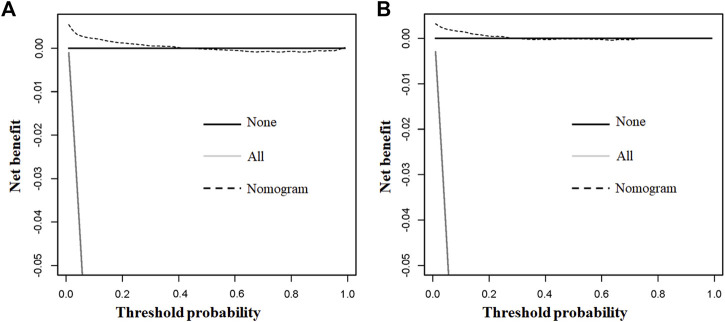
The training and the validation sets’ decision curve analysis (DCA) of the nomogram in our study for predicting bone metastasis in head and neck cancer patients. Black line denotes none; grey line all; dotted line nomogram.

The above eight independent risk variables obtained from multivariate logistic regression analysis were used to create a diagnostic nomogram. (Figure 1). By assigning values to related variables and calculating the total score of patients, the probability of BM in HNC patients was obtained. The training and the validation sets’ calibration curves showed a relatively similar agreement between the actual probability of BM and the predicted results (Figure 2). The AUC values of the training and the validation sets’ ROC curves were 0.893 and 0.850, respectively (Figure 3). At the same time, ROC curves also revealed that in the training and validation sets, the AUCs of all independent risk predictors were lower than that of the diagnostic nomogram (Figure 4). Furthermore, DCA curves demonstrated that the diagnostic nomogram had a high clinical application value and was an effective tool for evaluating and diagnosing the risk of BM in newly diagnosed HNC patients (Figure 5).

## Discussion

HNC patients’ OS rate and survival time increase as diagnosis and treatment technology improve. However, due to the inherent malignancy of HNC, the prolongation of its survival period inevitably increases the risk of distant metastases. For such distant metastases, there is currently no effective treatment. According to the study of Calhoun *et al.*, the average time from the diagnosis of any part of the distant metastases to the death of the patient was 4.3–7.3 months, and 86.7% of those patients would die within 12 months (Calhoun et al., 1994), while Bhandari and Jain proposed that the survival time did not exceed 8 months (Bhandari and Jain, 2013). Bone metastasis is involved in approximately 2–4% of HNC patients and 20–40% of HNC patients with distant metastases, making it one of the three most prevalent types of distant metastases (Pietropaoli et al., 2000). However, the low incidence of distant metastases and the lack of obvious early symptoms in most patients is easy to overlook or miss. By the time apparent symptoms appeared, such as those causing extreme pain, pathological fractures, and spinal cord compression, the patient was likely to have progressed to an advanced stage or to have developed multisite metastasis, thus lost the best treatment chance and failed to prolong OS even with radiotherapy and chemotherapy (Peters et al., 2015). Meanwhile, BM is prone to the thoracolumbar spine, and once paraplegia occurs, it will seriously affect the survival quality of patients (Bhandari and Jain, 2013).

Suzuki *et al.* analyzed the specific pattern of BM in head and neck squamous cell carcinoma (HNSCC) and concluded that patients with bone exclusive and single BM had a considerably higher median survival time than multiple organs and polyostotic metastases significantly (Suzuki et al., 2020). Sakisuka *et al.* also showed that early single BM in HNC had a longer survival time than those with multiple BM (Sakisuka et al., 2021). Thus, effective early prediction and intervention for distant metastases in HNC patients is expected to lead to better survival. Although the use of ^18^F-FDG PET/CT has dramatically improved the chances of detecting distant metastases, especially lung and bone metastases, the high cost limits its practical implementation, and the number of patients who can benefit is also limited. Deurvorst *et al.* used ^18^F-FDG PET/CT to screen distant metastases in 190 HNC patients with high-risk distant metastases. The results showed only a 12% positive detection rate, with sensitivity and negative predictive values of 46.2% and 82.6%, respectively. It could be seen that in HNSCC patients with high-risk distant metastases, ^18^F-FDG PET/CT exhibited a low sensitivity and a high negative predictive value for the detection of distant metastases in long-term follow-up (Deurvorst et al., 2018). The use of genomics and proteomics techniques and radiomics to evaluate the molecular characteristics of the primary tumor can also help predict the occurrence of BM (Han et al., 2021).

However, using these biomarkers to clinical decision-making right away is difficult and impractical, especially for newly diagnosed HNC patients (de Bree et al., 2018). In addition, Duprez *et al.* concluded that HPV negativity, positive lymph nodes, extra-nodal extension, increased N grade, and advanced tumor stage were associated with the development of distant metastases (Duprez et al., 2017). Other studies have shown that increased T grade, lesions located in the oropharynx, hypopharynx, and supraglottic, lymph nodes larger than 6 cm, local tumor recurrence, or second primary tumor were also risk factors for distant metastases in HNC patients (de Bree et al., 2000; Loh et al., 2005; Peters et al., 2015). The different conclusions reached in these studies may also be related to the bias in patient selection and the small sample size.

However, no studies have developed a diagnostic model for the risk of BM in newly diagnosed HNC patients, meaning that the risk of BM in individual patients cannot be assessed by integrating all independent BM-related risk indicators. In order to address this issue, we used a population-based database to identify independent risk predictors for BM in HNC patients and created a diagnostic nomogram based on demographic and tumor features to assess and predict the risk of BM in those newly diagnosed HNC patients. The discriminatory power of the diagnostic nomogram had been proved to be higher than that of any single predictor, indicating the importance of using an integrated diagnostic model. The present study identified race, primary site, tumor grade, T stage, N stage, and distant metastases (brain, liver, and lung) as independent risk predictors of BM development by analyzing 39,561 HNC patients from the SEER database. Furthermore, based on these eight risk factors obtained, we constructed a diagnostic nomogram to predict BM’s risk in HNC patients. According to reports, only a limited percentage of patients were diagnosed with BM simultaneously as the initial diagnosis of HNC, and most patients were found BM only when there were more obvious skeletal-related events in the subsequent course of the disease. The median time between confirmed BM and the initial diagnosis of HNC was approximately 11.5 months, leading to the progression of the distant metastases and the loss of treatment opportunities (Peters et al., 2015). With this nomogram, it is possible to predict the risk of BM for each newly diagnosed HNC patient simultaneously by simply assigning a value to a specific patient based on the variable information on the nomogram and calculating the total score, thus allowing for early intervention and individualized management of the risk, rather than waiting until the onset of typical skeletal-related events to intervene.

As in previous studies, age and gender were not risk factors for BM in HNC patients (Duprez et al., 2017). In contrast, the T and N stages at initial diagnosis were strongly associated with the probability of developing distant metastases. Specifically, a lower T stage was associated with a reduced prevalence of distant metastases, while a higher T stage was associated with a lower distant control rate of distant metastases. Lymph node-positive patients had a lower distant control rate than lymph node-negative patients (Duprez et al., 2017). Similar conclusions were obtained in our study, especially in HNC patients with the N3 stage, probably because cancer cells can invade surrounding tissues, capillaries, and lymphatic vessels, have more robust growth potential, and thus predisposing them to early metastasis. Our study identified the primary site as independent BM-related risk predictors in HNC patients, which showed that tumor biology features played an important role in disease progression and were linked to the onset and progression of BM. Kotwall *et al.* performed autopsies on 832 HNC patients and found the distant metastases’ prevalence in the hypopharynx HNC were as high as 60% (Kotwall et al., 1997). Our study also found that the larger tumor grade at the initial presentation, brain, lung, and liver metastases were independent risk factors for BM in HNC patients. Patients with grade IV cancer had the highest rate of distant metastases at the time of diagnosis (Kotwall et al., 1997). Although we found that HNC patients with brain, lung, and liver metastases were at higher risk for BM, the complex mechanisms behind this are still not well understood, and studies on whether metastases occur sequentially have not been reported and whether metastases from other sites contribute to the development of BM synergistically is also worthy of further study. Furthermore, while race as a risk factor for distant metastases is not as intuitive as the primary site, histology type, T stage, and N stage, the race is a risk factor for distant metastases in other cancers with some specificity. For example, white patients with HNC or thyroid cancer are more likely to develop BM than black patients. In contrast, Asian and Pacific Islander lung cancer patients have a higher probability of BM than whites, which may be related to socioeconomic status and specific biological factors, such as the high prevalence of SCC in white patients (Schwartz et al., 2003; Tong et al., 2020; Xu et al., 2020; Chi et al., 2021).

Our study has several advantages; first, to our knowledge, this is the first diagnostic nomogram used to predict the BM of HNC patients. The model was constructed based on a population with a sufficiently large enough sample size to cover almost all kinds of HNC, guaranteeing the representativeness and clinical value of the study results. Second, by performing ROC analysis on the independent risk factors with the constructed diagnostic nomogram, we found that the discriminative power of any independent risk factors was inferior to the integrated diagnostic nomogram, showing the superiority of the integrated predictive power of the diagnostic nomogram. Then, compared with the genetic and molecular level markers associated with BM, the independent risk factors identified in our study were readily available in daily practice, allowing for easy manipulation and personalized prediction.

However, our research inevitably has several limitations. First, the limited number of HNC-BM patients (*n* = 332) may cause potential errors. Secondly, it is a retrospective study, and selection bias is inevitable. Then, although it contains several of the most common metastatic sites in HNC patients, it lacks information on the order and severity of metastases and other critical locations of potential metastases, such as skin and pleura, which are also common metastatic sites for HNC. Finally, more data from other research centers for external verification will improve the applicability and accuracy of our diagnostic nomogram.

## Conclusion

In conclusion, our study showed that race, primary site, tumor grade, T stage, N stage, and distant metastases (brain, liver, and lung) were independent risk factors for BM in HNC patients. The diagnostic nomogram constructed using the above risk factors could quickly determine the probability of BM in newly diagnosed HNC patients, assist doctors in providing personalized management of HNC patients, especially in HNC patients with potentially high-risk BM, and conduct better early education, early detection, and early diagnosis and treatment, to maximize the benefits of patients.

## Data Availability

Publicly available datasets were analyzed in this study. This data can be found here: SEER dataset repository (https://seer.cancer.gov/).

## Abbreviations

AUC, area under the curve; BM, bone metastasis; DCA, decision curve analysis; HNC, head and neck cancer; HNSCC, head and neck squamous cell carcinoma; SEER, Surveillance, epidemiology and end results; SCC, squamous cell carcinoma; OS, overall survival; ROC, receiver operating characteristic.

## References

[B1] Bhandari V. Jain R. (2013). A Retrospective Study of Incidence of Bone Metastasis in Head and Neck Cancer. J. Can. Res. Ther. 9 (1), 90–93. 10.4103/0973-1482.110385 23575081

[B2] Calhoun K. H. Fulmer P. Weiss R. Hokanson J. A. (1994). Distant Metastases from Head and Neck Squamous Cell Carcinomas. Laryngoscope 104 (10), 1199–1205. 10.1288/00005537-199410000-00003 7934588

[B3] Carvalho A. L. Nishimoto I. N. Califano J. A. Kowalski L. P. (2005). Trends in Incidence and Prognosis for Head and Neck Cancer in the United States: a Site-specific Analysis of the SEER Database. Int. J. Cancer 114 (5), 806–816. 10.1002/ijc.20740 15609302

[B4] Chi C. Fan Z. Yang B. Sun H. Zheng Z. (2021). The Clinical Characteristics and Prognostic Nomogram for Head and Neck Cancer Patients with Bone Metastasis. J. Oncol. 2021, 1–12. 10.1155/2021/5859757 PMC8490031 34616453

[B5] de Bree R. Deurloo E. E. Snow G. B. Leemans C. R. (2000). Screening for Distant Metastases in Patients with Head and Neck Cancer. Laryngoscope 110 (3 Pt 1), 397–401. 10.1097/00005537-200003000-00012 10718426

[B6] de Bree R. Senft A. Coca-Pelaz A. Kowalski L. Lopez F. Mendenhall W. (2018). Detection of Distant Metastases in Head and Neck Cancer: Changing Landscape. Adv. Ther. 35 (2), 161–172. 10.1007/s12325-018-0662-8 29396680

[B7] Deurvorst S. E. Hoekstra O. S. Castelijns J. A. Witte B. I. Leemans C. R. de Bree R. (2018). Clinical Value of 18 FDG PET/CT in Screening for Distant Metastases in Head and Neck Squamous Cell Carcinoma. Clin. Otolaryngol. 43 (3), 875–881. 10.1111/coa.13074 29377508

[B8] Duprez F. Berwouts D. De Neve W. Bonte K. Boterberg T. Deron P. (2017). Distant Metastases in Head and Neck Cancer. Head Neck 39 (9), 1733–1743. 10.1002/hed.24687 28650113

[B9] Guo K. Xiao W. Chen X. Zhao Z. Lin Y. Chen G. (2021). Epidemiological Trends of Head and Neck Cancer: A Population-Based Study. BioMed Res. Int. 2021, 1–14. 10.1155/2021/1738932 34337000 PMC8294963

[B10] Han Y. Sui Z. Jia Y. Wang H. Dong Y. Zhang H. (2021). Metastasis Patterns and Prognosis in Breast Cancer Patients Aged ≥ 80 years: a SEER Database Analysis. J. Cancer 12 (21), 6445–6453. 10.7150/jca.63813 34659535 PMC8489144

[B11] Kotwall C. Sako K. Razack M. S. Rao U. Bakamjian V. Shedd D. P. (1997). Metastatic Patterns in Squamous Cell Cancer of the Head and Neck. Am. J. Surg. 154, 439–442. 10.1016/0002-9610(89)90020-2 3661849

[B12] Lee D. H. Kim M. J. Roh J.-L. Kim S.-B. Choi S.-H. Nam S. Y. (2012). Distant Metastases and Survival Prediction in Head and Neck Squamous Cell Carcinoma. Otolaryngol. Head. Neck Surg. 147 (5), 870–875. 10.1177/0194599812447048 22581637

[B13] Lin Z. Yan S. Zhang J. Pan Q. (2018). A Nomogram for Distinction and Potential Prediction of Liver Metastasis in Breast Cancer Patients. J. Cancer 9 (12), 2098–2106. 10.7150/jca.24445 29937928 PMC6010683

[B14] Loh K. S. Brown D. H. Baker J. T. Gilbert R. W. Gullane P. J. Irish J. C. (2005). A Rational Approach to Pulmonary Screening in Newly Diagnosed Head and Neck Cancer. Head. Neck 27 (11), 990–994. 10.1002/hed.20261 16136584

[B15] Mourad M. Jetmore T. Jategaonkar A. A. Moubayed S. Moshier E. Urken M. L. (2017). Epidemiological Trends of Head and Neck Cancer in the United States: A SEER Population Study. J. Oral Maxillofac. Surg. 75 (12), 2562–2572. 10.1016/j.joms.2017.05.008 28618252 PMC6053274

[B16] Peters T. T. Senft A. Hoekstra O. S. Castelijns J. A. Witte B. I. Leemans C. R. (2015). Pretreatment Screening on Distant Metastases and Head and Neck Cancer Patients: Validation of Risk Factors and Influence on Survival. Oral Oncol. 51 (3), 267–271. 10.1016/j.oraloncology.2014.12.006 25552384

[B17] Pietropaoli M. P. Damron T. A. Vermont A. I. (2000). Bone Metastases from Squamous Cell Carcinoma of the Head and Neck. J. Surg. Oncol. 75 (2), 136–140. 10.1002/1096-9098(200010)75:2<136:aid-jso11>3.0.co;2-d 11064394

[B18] Riaz A. Ali A-R. Eleanor C. Naeem D. Christos I. Ginimol M. (2019). STROCSS 2019 Guideline: Strengthening the Reporting of Cohort Studies in Surgery. Int. J. Surg. 72, 156–165. 31704426 10.1016/j.ijsu.2019.11.002

[B19] Sakisuka T. Kashiwagi N. Doi H. Takahashi H. Arisawa A. Matsuo C. (2021). Prognostic Factors for Bone Metastases from Head and Neck Squamous Cell Carcinoma: A Case Series of 97 Patients. Mol. Clin. Oncol. 15 (5), 246. 10.3892/mco.2021.2408 34650813 PMC8506565

[B20] Sarfraz H. Gentille C. Ensor J. Wang L. Wong S. Ketcham M. S. (2021). Primary Cutaneous Anaplastic Large‐cell Lymphoma: a Review of the SEER Database from 2005 to 2016. Clin. Exp. Dermatol 46 (8), 1420–1426. 10.1111/ced.14777 34081802

[B21] Schwartz K. L. Crossley‐May H. Vigneau F. D. Brown K. Banerjee M. (2003). Race, Socioeconomic Status and Stage at Diagnosis for Five Common Malignancies. Cancer Causes Control 14 (8), 761–766. 10.1023/a:1026321923883 14674740

[B22] Sung H. Ferlay J. Siegel R. L. Laversanne M. Soerjomataram I. Jemal A. (2021). Global Cancer Statistics 2020: GLOBOCAN Estimates of Incidence and Mortality Worldwide for 36 Cancers in 185 Countries. CA A Cancer J. Clin. 71 (3), 209–249. 10.3322/caac.21660 33538338

[B23] Suzuki A. Kashiwagi N. Doi H. Ishii K. Doi K. Kitano M. (2020). Patterns of Bone Metastases from Head and Neck Squamous Cell Carcinoma. Auris Nasus Larynx 47 (2), 262–267. 10.1016/j.anl.2019.08.001 31445714

[B24] Tadros M. Mago S. Miller D. Ungemack J. A. Anderson J. C. Swede H. (2021). The Rise of Proximal Colorectal Cancer: a Trend Analysis of Subsite Specific Primary Colorectal Cancer in the SEER Database. aog 34 (4), 559–567. 10.20524/aog.2021.0608 PMC8276357 34276196

[B25] Tong Y. Hu C. Huang Z. Fan Z. Zhu L. Song Y. (2020). Novel Nomogram to Predict Risk of Bone Metastasis in Newly Diagnosed Thyroid Carcinoma: a Population-Based Study. BMC Cancer 20 (1), 1055. 10.1186/s12885-020-07554-1 33143688 PMC7607856

[B26] Xu G. Cui P. Zhang C. Lin F. Xu Y. Guo X. (2020). Racial Disparities in Bone Metastasis Patterns and Targeted Screening and Treatment Strategies in Newly Diagnosed Lung Cancer Patients. Ethn. Health 27, 329–342. 10.1080/13557858.2020.1734775 32223328

[B27] Zhou C. Zhang Y. Hu X. Fang M. Xiao S. (2021). The Effect of Marital and Insurance Status on the Survival of Elderly Patients with Stage M1b Colon Cancer: a SEER-Based Study. BMC Cancer 21 (1), 891. 10.1186/s12885-021-08627-5 34353300 PMC8340368

