# Supplementary material for: A Nomogram for Predicting the Risk of Bone Metastasis in Newly Diagnosed Head and Neck Cancer Patients: A Real-World Data Retrospective Cohort Study From SEER Database
Source: Front Genet. 2022 May 30;13:865418. doi: 10.3389/fgene.2022.865418 (PMC9189363; doi:10.3389/fgene.2022.865418)
Supplement: Supplementary file 1 [file DataSheet1.docx]

**Table S1.** The detailed scores of independent risk predictors of bone metastasis (BM) in the diagnostic nomogram.

| **BM-related variables** | **Corresponding score assignments in nomogram** |
| --- | --- |
| **Race** | |
| Black | 16 |
| White | 1 |
| Other | 1 |
| **Primary site** | |
| Oral cavity | 16 |
| Lip | 2 |
| Oropharynx | 13 |
| Nasopharynx | 58 |
| Hypopharynx | 8 |
| Salivary Gland | 35 |
| Sinonasal | 43 |
| Larynx | 0 |
| **Tumor grade** | |
| Ⅰ | 16 |
| Ⅱ | 35 |
| Ⅲ | 54 |
| Ⅳ | 48 |
| **T Stage** | |
| T1 | 16 |
| T2 | 16 |
| T3 | 32 |
| T4 | 48 |
| **N Stage** | |
| N0 | 16 |
| N1 | 41 |
| N2 | 40 |
| N3 | 50 |
| **Lung metastasis** | |
| Absent | 16 |
| Present | 89 |
| **Liver metastasis** | |
| Absent | 16 |
| Present | 100 |
| **Brain metastasis** | |
| Absent | 16 |
| Present | 80 |
